# Histological Diagnostic Yield and Clinical Significance of the First Biopsy in Device-Assisted Enteroscopy in Patients with Small Bowel Diseases: A KASID Multicenter Study

**DOI:** 10.3390/diagnostics12040964

**Published:** 2022-04-12

**Authors:** Hyeon Jeong Goong, Tae Joon Kim, Kwangwoo Nam, Jihye Park, Jin-Oh Kim, Hyun Gun Kim, Bong Min Ko, Seong Ran Jeon, Small Intestine Research Group of the Korean Association for the Study of Intestinal Diseases (KASID)

**Affiliations:** 1 Department of Internal Medicine, Soonchunhyang University College of Medicine, Bucheon 14584, Korea; goong@schmc.ac.kr (H.J.G.); kopa9445@schmc.ac.kr (B.M.K.); 2 Department of Internal Medicine, Sungkyunkwan University School of Medicine, Seoul 06351, Korea; tj23.kim@samsung.com; 3 Department of Internal Medicine, Dankook University Hospital, Dankook University College of Medicine, Cheonan 31116, Korea; nambag@naver.com; 4 Department of Internal Medicine, Yonsei University College of Medicine, Seoul 03722, Korea; mint860925@gmail.com; 5 Department of Internal Medicine, Soonchunhyang University College of Medicine, Seoul 04401, Korea; jokim@schmc.ac.kr (J.-O.K.); medgun@schmc.ac.kr (H.G.K.)

**Keywords:** device assisted enteroscopy, small bowel, biopsy

## Abstract

Device-assisted enteroscopy (DAE) enables the direct visualization of small bowel lesions with histological diagnosis; however, few studies have described the diagnostic performance of enteroscopic biopsy. We investigated the diagnostic performance of enteroscopic biopsy. We used a nationwide multicenter enteroscopy database to identify patients who underwent DAE with biopsy for small bowel diseases. The patients were classified into the tumor and non-tumor groups according to the final diagnosis. They were also divided into diagnostic and non-diagnostic groups based on the enteroscopic biopsy results. The clinical significance of the first biopsy and histological diagnostic yield of DAE were analyzed. Among the 112 procedures investigated, 32 (28.9%) were diagnosed with tumors, and 80 (71.7%) were diagnosed with non-tumor diseases. The overall histological diagnostic yield of DAE was 43.7%. The histological diagnostic yield was significantly higher in the tumor than in the non-tumor group (81.2% vs. 28.8%, *p* < 0.001). The mean number of biopsies was significantly higher in the diagnostic than in the non-diagnostic group (5.6 ± 3.3 vs. 3.7 ± 2.1, *p* = 0.001). In the diagnostic group, 87.7% of the cases were histologically confirmed at the first biopsy. Therefore, the first biopsy should be performed carefully.

## 1. Introduction

The development of capsule endoscopy (CE) and device-assisted enteroscopy (DAE) has increased the diagnostic yield (DY) for small bowel disease. Accordingly, histological diagnosis using DAE is becoming important. DAE enables endoscopic procedures (e.g., biopsy, polypectomy, and hemostasis) and direct visualization of lesions. The DY of DAE for small bowel diseases has been reported to range from 47% to 90% [1,2,3,4,5,6,7]. However, previous data on the DY of DAE included both endoscopic and histological diagnoses. In addition, as the most common indication for DAE in those studies was suspected to be small bowel bleeding (SSBB), the DY was mainly evaluated on the basis of endoscopic diagnosis rather than histological diagnosis. Therefore, studies on the diagnostic performance of enteroscopic biopsy are limited. The optimal biopsy strategies in conventional endoscopy have been investigated. Theoretically, the diagnostic accuracy is expected to improve as the number of biopsy specimens increases. However, Choi et al. reported that 78.1–81.3% of histological diagnoses were confirmed at the first biopsy in patients with advanced gastric and colon cancers. Additionally, the DY increased to 93.8–98.3% until the fourth biopsy but did not significantly increase further with more biopsies [8]. Therefore, the quality of the first biopsy is important for diagnosis. In addition, the effect of changes in the lesion surface (e.g., bleeding) on subsequent biopsies might limit the improvement in DY with increasing number of biopsies. Although DAE is relatively safe with a low rate of procedure-related complications, it is technically difficult and more invasive than conventional endoscopy. Therefore, the technical difficulties associated with DAE may affect the diagnostic efficacy of biopsy. However, the histological performance of enteroscopic biopsy and the role of the first biopsy have not been thoroughly investigated. Therefore, in this study, we aimed to evaluate the histological DY of DAE, the factors affecting the DY of enteroscopic biopsy, and the clinical significance of the first biopsy.

## 2. Materials and Methods

### 2.1. Study Design and Patients

Using a nationwide multicenter enteroscopy database, we retrospectively analyzed the clinical data and medical records of patients who underwent DAE with biopsy for the evaluation of small bowel diseases between March 2004 and August 2021. We excluded cases in which the final diagnosis could not be confirmed by histopathology or in which the details of biopsy profiles were not assessable. This study was performed in accordance with the ethical principles of the Declaration of Helsinki and was approved by the institutional review board of each participating institution. The requirement for informed consent was waived by the institutional review boards owing to the retrospective nature of this study.

### 2.2. Device Assisted Enteroscopy

To identify the location and characteristics of small-bowel lesions, capsule endoscopy, computed tomography (CT)/CT enterography (CTE), magnetic resonance enterography (MRE), or small-bowel follow-through was performed before DAE. The route of entry (antegrade or retrograde insertion) was determined by each endoscopist according to the suspected lesion location, as assessed on prior imaging studies. All patients were instructed to fast for at least 8 h before the procedure. For the retrograde approach, the patients underwent bowel preparation with 2 L polyethylene glycol electrolyte lavage solution before the procedure. Most patients underwent DAE under moderate conscious sedation with midazolam, pethidine, or propofol. DAE was performed using a single-balloon enteroscopy (SBE) system (SIF-Q180; Olympus Medical Inc., Tokyo, Japan) or a double-balloon enteroscopy (DBE) system (EN-450P5/20, T5/20; Fujinon Inc., Saitama, Japan), depending on the available equipment at each institution. Enteroscopic biopsy was performed using the biopsy forceps available for enteroscopy at each facility.

### 2.3. Data Collection and Outcome Measures

Patient-related variables, including age, sex, and history of abdominal surgery, were reviewed. The indications for DAE included SSBB, imaging abnormalities detected before DAE, symptoms or signs of suspected small-bowel disease, and suspected/established Crohn’s disease (CD). Lesion-related variables, including location and histopathological results, were reviewed using endoscopic images and histopathological reports. The final diagnosis was confirmed by histopathological examination of enteroscopic biopsy, conventional endoscopic biopsy, or surgical specimens. The histopathological diagnosis was confirmed by expert pathologists at each hospital. The final diagnosis of CD and vasculitis was confirmed using a combination of clinical, endoscopic, radiologic, laboratory, and histological findings. The patients were classified into two groups according to the final diagnosis: tumor and non-tumor groups. We also divided the patients into the diagnostic and non-diagnostic groups based on the result of enteroscopic biopsy relative to the final diagnosis. The diagnostic group was defined as patients in whom the enteroscopic biopsy result was concordant with the final diagnosis. With respect to procedure-related factors, the DAE system, insertion route, number of biopsies, and post-procedural complications (e.g., perforation, bleeding, and pancreatitis) were documented.

The primary outcome of this study was the clinical significance of the first biopsy for the diagnosis of small bowel diseases. The secondary outcomes were the histological DY of DAE and the factors affecting the diagnostic performance of enteroscopic biopsy.

### 2.4. Statistical Analysis

Categorical variables are presented as frequencies and percentages. Continuous variables are presented as mean ± standard deviation values. All categorical and continuous variables were compared using the chi-square test and Student’s t-test, respectively. Fisher’s exact test was used when the expected value in any of the cells of a contingency table was <5. A logistic regression model with odds ratios (ORs) and 95% confidential intervals (CIs) was used to evaluate the factors associated with the DY of enteroscopic biopsy. A two-sided *p* value of <0.05 was considered statistically significant. Statistical analysis was performed using SPSS (version 22.0; SPSS Inc., Chicago, IL, USA).

## 3. Results

### 3.1. Baseline Characteristics of Patients and Small Bowel Lesions

**Figure 1:**
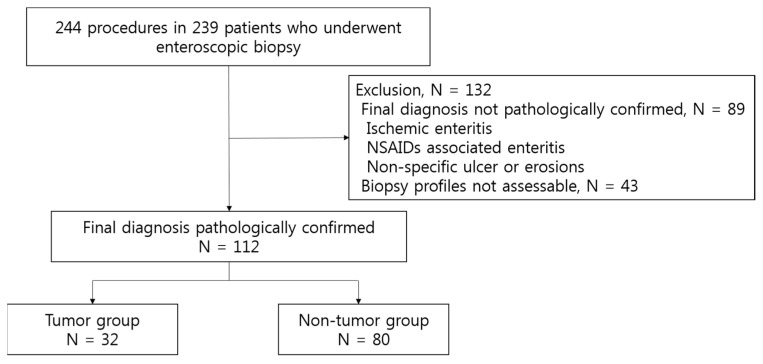
Flow diagram for selection the screening-eligible case.

Among the 244 procedures performed in 239 patients, histological confirmation of the final diagnosis was not obtained in 89 patients. These patients had a clinical diagnosis of ischemic enteritis, non-steroidal anti-inflammatory drug-associated enteropathy, or non-specific enteritis with non-specific inflammation on enteroscopic biopsy. Additionally, detailed biopsy profiles were not assessable in 43 patients. Therefore, 112 procedures in 107 patients were included in the final analysis (Figure 1). The baseline characteristics of the patients and lesions are summarized in Table 1. The most common indications for DAE were imaging abnormalities detected before DAE (28.6%), gastrointestinal symptoms or signs (27.7%), and suspected/established CD (25.9%). In terms of the final diagnosis, tumors were diagnosed in 32 patients (28.9%), and non-tumor diseases were diagnosed in 80 patients (71.4%). Imaging studies of the location and characteristics of the lesions were mostly performed using CT/CTE or MRE (87.5%) before enteroscopy. Of the patients, 70.5% were treated with medical therapy, 25.9% underwent surgery, and 1.8% underwent endoscopic treatment. Two patients diagnosed with CD underwent endoscopic balloon dilatation.

### 3.2. DAE and Enteroscopic Biopsy in the Tumor and Non-Tumor Groups

The procedure profiles of DAE and enteroscopic biopsy according to the final diagnosis are shown in Table 2. No significant difference in enteroscopy methods was observed between the tumor and non-tumor groups, and DBE was the most frequently used method (96.4%). The proportion of lesions located in the ileum was significantly higher in the non-tumor group than in the tumor group (*p* < 0.001). In this regard, the proportion of retrograde insertions was significantly higher in the non-tumor group (*p* < 0.001). Lesions detected in two or more segments of the small intestine were diagnosed as B-cell lymphoma in the tumor group (n = 1) and CD (n = 5) and vasculitis (n = 1) in the non-tumor group. The total number of biopsies was higher in the tumor group than in the non-tumor group, although the difference was not significant (5.2 ± 3.4 vs. 4.3 ± 2.6, *p* = 0.118). The total procedure time was significantly shorter in the tumor group than in the non-tumor group (49.0 ± 29.2 vs. 75.1 ± 43.0 min, *p* = 0.003). Procedure-related complications such as bleeding, pancreatitis, and perforation did not occur in any of the cases.

### 3.3. Clinical Significance of the First Biopsy

**Figure 2:**
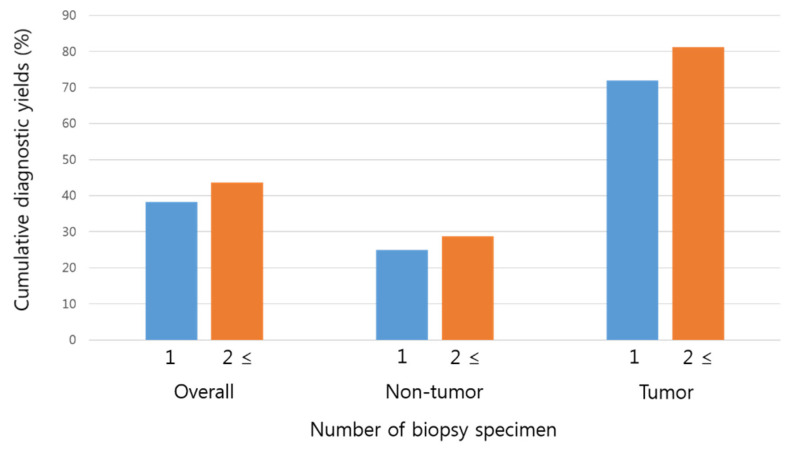
Cumulative diagnostic yields according to the number of serial enteroscopic biopsies.

Figure 2 shows the cumulative DYs at the first and second or more biopsies. The overall DY of the first biopsy was 38.3%. After two or more biopsies, the cumulative DY increased to 43.7%, without statistical significance (*p* = 0.415). Among patients in whom diagnosis was made by enteroscopic biopsy, histological confirmation was obtained at the first biopsy in 87.7% and at the second or further biopsies in 12.3%. In subgroup analysis, the DY increased after two or more biopsies, although the difference was not statistically significant in both the non-tumor (from 25% to 28.8%, *p* = 0.593) and tumor (from 71.9% to 81.2%, *p* = 0.376) groups.

### 3.4. Histological DY of DAE and Factors Affecting the DY

The overall DY of enteroscopic biopsy was 43.7% (49/112). The DYs according to enteroscopic procedures and lesion characteristics are shown in Table 3. The patients were classified into the diagnostic (n = 49) and non-diagnostic (n = 63) groups. No significant associations were found between the enteroscopy methods, history of abdominal surgery, or diagnostic information obtained on prior imaging studies and the DY of enteroscopic biopsy. However, in the diagnostic group, the proportion of lesions in the duodenum or jejunum was higher than that of lesions in the ileum or in multiple sites of the small bowel (65.2% vs. 28.8%, *p* < 0.001). In terms of the insertion route, the proportion of anterograde insertion was significantly higher than that of retrograde insertion in the diagnostic group (55.7% vs. 29.4%, *p* = 0.005). The mean number of biopsies was significantly higher in the diagnostic group than in the non-diagnostic group (5.6 ± 3.3 vs. 3.7 ± 2.1, *p* = 0.001). The DY was significantly higher in the tumor group than in the non-tumor group (81.2% vs. 28.8%, *p* < 0.001). In the non-tumor group, no significant difference in DY was observed between CD and non-CD cases (27.1% vs. 33.3%, *p* = 0.589). The DYs for all diseases are shown in Supplementary Table S1. In univariate and multivariable logistic regression analyses, tumors (OR, 6.21; 95% CI, 1.80–21.43) and a higher number of biopsies (OR, 1.36; 95% CI, 1.13–1.65) were significant factors for a positive DY (Table 4).

## 4. Discussion

As the DY and therapeutic yield of enteroscopy have improved with advances in endoscopic technology, the diagnostic accuracy of enteroscopic biopsy is expected to not significantly differ from that of conventional endoscopy. In addition, we considered that the biopsy-targeting technique during DAE may be comparable to that during conventional endoscopy. Therefore, we hypothesized that the significance of the first biopsy during DAE would be similar to that previously reported for conventional endoscopy. However, whether the technical difficulty of DAE causes a difference in biopsy performance compared with conventional endoscopy has not yet been investigated.

In this study, although the number of biopsies was a significant factor for DY, 87.7% of the cases histologically diagnosed by DAE were confirmed at the first biopsy in both the tumor and non-tumor groups. The quality of the first biopsy is important because post-biopsy bleeding interferes with the endoscopic visual field during the subsequent biopsies. In tumors, optical investigation of the surface pattern before biopsy is necessary to avoid sampling of the necrotic surface or of the precancerous mucosa near the cancer-altered mucosa. In inflammatory diseases, exudate, debris, or necrosis-covered mucosa usually interferes with biopsy targeting. In addition, endoscopists might hesitate about performing further biopsy of ulcerative or erosive surfaces because of concerns about bleeding or perforation. The significance of an adequate first biopsy has been shown in previous studies on optimal biopsy strategies for diagnosing tumors during conventional endoscopy. Choi et al. analyzed the diagnostic accuracy of endoscopic biopsy in 59 patients with advanced gastric cancer and 32 patients with advanced colon cancer [8]. The histological DY of the first biopsy was 81.3% for gastric cancer and 78.1% for colon cancer. Although the second to fourth biopsies showed improved diagnostic accuracy of up to 98.3%, further biopsies did not significantly affect the DY. In addition, a retrospective study of 858 cases of early gastric cancer demonstrated that the diagnostic sensitivity of one endoscopically obtained biopsy specimen was 83.9% [9]. With advances in endoscopic imaging resolution and instrumentation, accurate biopsy targeting has become possible, resulting in appropriate tissue acquisition with fewer biopsies than in the past. The histological DY of the first biopsy in our study was similar to that in previous reports. Therefore, the quality of the first biopsy is also important in DAE.

Studies on the diagnostic accuracy of enteroscopic biopsy are limited. In a multicenter study that analyzed 877 DAE procedures, among 294 patients who underwent enteroscopic biopsy, the histological DY was 59.5% for overall small bowel diseases, 72.9% for tumors, and 54.5% for ulcer/inflammatory/stricture diseases [10]. In our study, the overall histological DY was 43.7%. The DY according to the two disease groups was 81.2% in the tumor group and 28.8% in the non-tumor group (*p* < 0.001). This difference in DY between the tumor and non-tumor groups was similar to that of biopsy during conventional endoscopy. For tumors, the histological diagnosis rate was reported to be 80–90% in conventional enteroscopy [11,12,13,14,15]. Therefore, in this study, enteroscopic biopsy showed comparable diagnostic effectiveness to conventional endoscopic biopsy in patients with tumors. With respect to non-tumor diseases, the histological diagnosis rate in our study was lower than that for tumors. Previous reports have shown a poor correlation between endoscopic appearance and histological results in inflammatory disease in small bowel [16,17]. Tun et al. reported that histology was diagnostic in 8–15% of patients with suspected small bowel CD. In addition, 58% of the cases with a macroscopically positive appearance on DAE showed histologically normal or non-specific findings [18]. In addition, histological confirmation is difficult in other inflammatory diseases because of the discontinuous involvement of inflammation. The histological diagnosis rate was 20–38% for intestinal tuberculosis and 5% for vasculitis [19,20]. The histological detection rate of eosinophilic gastroenteritis was estimated to be up to 80% [21]. Similar to the results of previous studies, CD, intestinal tuberculosis, and vasculitis showed a lower histological diagnosis rate (<50%) than eosinophilic gastroenteritis (60%) in the current study.

In this study, we found that an increase in the number of biopsies improved the histological DY in logistic regression analysis (OR, 1.36; *p* = 0.001). The mean number of biopsies in the tumor group was higher than that in the non-tumor group, although the difference was not statistically significant (*p* = 0.118). Current guidelines recommend an adequate number of biopsies for tumors (four to six biopsies) during conventional endoscopy [22,23,24,25,26]. However, more biopsies are required to diagnose inflammatory diseases. The histological diagnosis of CD requires serial biopsy of multiple segments of normal and abnormal mucosa [27]. At least eight biopsies are recommended for diagnosing intestinal tuberculosis [20]. Even for eosinophilic gastroenteritis, which has a relatively higher histological diagnosis rate than other inflammatory diseases, more than five to six biopsies are recommended [21]. Therefore, considering the current recommendations, the number of biopsies in the non-tumor group in this study was relatively low. Moreover, we could not determine whether the number of biopsies was significantly different between the diagnostic and non-diagnostic groups among patients with non-tumor diseases (5.3 ± 3.2 vs. 3.9 ± 2.2, *p* = 0.053). Unlike in conventional endoscopy, no recommendation on the optimal number of biopsies during DAE has been proposed. In this study, the mean number of biopsies in the tumor group was similar, and the DY was comparable, to that in conventional endoscopy. However, in the case of inflammatory diseases, the DY might have been underestimated because the mean number of biopsies was lower than the current recommendations for conventional endoscopy. As enteroscopy is more invasive and technically difficult than conventional endoscopy, further studies on the optimal number of enteroscopic biopsies for each small bowel disease are necessary.

This study had a few limitations. First, owing to the retrospective study design, the procedural protocols were inconsistent. Differences in the biopsy forceps, sedation protocols, endoscopists’ experiences, and skills in each participating institution might have affected the DY. However, we were unable to evaluate these factors in our study. Second, as discussed earlier, the number of biopsies for inflammatory diseases was lower than the current recommendations. The number of biopsies for inflammatory diseases was mostly fewer than five to six. In this regard, the DY was relatively lower than previously published data. Third, the DYs of SBE and DBE could not be compared because DBE was performed in 96.4% of the procedures. However, the technical success rate, DY, and therapeutic yield of SBE and DBE were not different in recent studies [28,29,30]. Despite these limitations, this study is valuable because data on biopsy protocols in DAE are lacking, compared with conventional endoscopy. Enteroscopic biopsy is technically challenging, and the anatomy of the small intestine is different from that of the stomach or colon. Therefore, a further large-scale prospective study investigating the diagnostic performance of enteroscopic biopsy is needed to develop adequate biopsy protocols according to disease type.

## 5. Conclusions

The histological DY of enteroscopic biopsy was comparable to that of conventional endoscopy. The DY was higher for tumors than for non-tumor diseases. In addition, the DY improved as the number of biopsies increased. However, most of the cases diagnosed by enteroscopic biopsy were histologically confirmed at the first biopsy. Therefore, care should be taken when performing the first biopsy.

## Figures and Tables

**Table 1:** Baseline characteristics of the cases and final diagnosis.

	N = 112
Age, year, mean ± SD ^1^	43.4 ± 16.4
Male, n (%)	82 (73.2)
History of abdominal surgery, n (%)	18 (16.1)
Indications for DAE ^2^, n (%)	
Suspected small bowel bleeding	29 (25.9)
Imaging abnormalities	32 (28.6)
Gastrointestinal symptoms and signs	31 (27.7)
Evaluation of suspected/established Crohn’s disease	14 (12.5)
Others (anemia, treatment, surveillance)	6 (5.3)
Prior diagnostic modalities, n (%)	
Capsule endoscopy	25 (22.3)
CT/CTE or MRE	98 (87.5)
SBFT ^3^	29 (25.9)
Final diagnosis, n (%)	
Tumor disease	32 (28.6)
Adenocarcinoma	15 (13.4)
Lymphoma	11 (9.8)
Malignant GIST ^4^	2 (1.8)
Leiomyosarcoma	2 (1.8)
Inflammatory polyp	1 (0.9)
Lipoma	1 (0.9)
Non-Tumor disease	80 (71.4)
Crohn’s disease	59 (52.7)
Intestinal tuberculosis	10 (8.9)
Eosinophilic enteritis	3 (2.7)
Behcet’s disease	3 (2.7)
Vasculitis	3 (2.7)
Meckel diverticulum	2 (1.8)
Treatment, n (%)	
Medical treatment	79 (70.5)
Endoscopic treatment	2 (1.8)
Surgical treatment	29 (25.9)
Observation	2 (1.8)

^1^ SD, standardized deviation; ^2^ DAE, device-assisted enteroscopy; ^3^ SBFT, small bowel follows through; ^4^ GIST, gastrointestinal stromal tumor.

**Table 2:** Technical characteristics of device-assisted enteroscopy and biopsy in tumor and non- tumor groups.

	Tumor (N = 32)	Non-tumor (N = 80)	*p* Value
Lesion location, n (%)			<0.001
Duodenum	3 (100)	0	
Jejunum	25 (58.1)	18 (41.9)	
Ileum	3 (5.1)	56 (94.9)	
Multiple	1 (14.3)	6 (85.7)	
Enteroscopy methods			0.556
SBE ^1^	0 (0)	3 (100)	
DBE ^2^	32 (29.4)	77 (70.6)	
Insertion route			<0.001
Anterograde	28 (45.9)	33 (54.1)	
Retrograde	4 (7.8)	47 (92.2)	
Total number of biopsy, mean ± SD ^3^	5.2 ± 3.4	4.3 ± 2.6	0.118
Total procedure time, minutes, mean ± SD	49.0 ± 29.2	75.1 ± 43.0	0.003
Complications, n (%)	0	0	NA ^4^

^1^ SBE, single balloon enteroscopy; ^2^ DBE, double balloon enteroscopy; ^3^ SD, standardized deviation; ^4^ NA, not applicable.

**Table 3:** Diagnostic yields of enteroscopic biopsy according to the technical characteristics of device assisted enteroscopy and final diagnosis.

	Diagnostic (N = 49)	Non-Diagnostic (N = 63)	*p*-Value
Lesion location, n (%)			<0.001
Duodenum/jejunum	30 (65.2)	16 (34.8)	
Ileum/multiple	19 (28.8)	47 (71.2)	
Matched diagnosis on prior imaging study, n (%)			
Capsule endoscopy	8 (36.4)	14 (63.6)	0.436
CT or MRI	34 (45.9)	40 (54.1)	0.513
History of abdominal surgery, n (%)			0.948
Yes	8 (44.4)	10 (55.6)	
No	41 (43.6)	53 (56.4)	
Enteroscopy methods, n (%)			0.99<
DBE	48 (44.0)	61 (56.0)	
SBE	1 (33.3)	2 (66.7)	
Insertion route, n (%)			0.005
Anterograde	34 (55.7)	27 (44.3)	
Retrograde	15 (29.4)	36 (70.6)	
Biopsy number, mean ± SD	5.6 ± 3.3	3.7 ± 2.1	0.001
Non-Tumor	5.3 ± 3.2	3.9 ± 2.2	0.053
Tumor	5.9 ± 3.4	2.5 ± 1.6	0.025
Final diagnosis, n (%)			<0.001
Non-tumor	23 (28.8)	57 (71.2)	
Crohn’s disease	16 (27.1)	43 (72.9)	
Non-Crohn’s disease	7 (33.3)	14 (66.7)	
Tumor	26 (81.2)	6 (18.8)	

**Table 4:** Logistic regression analysis of factors for diagnostic yields of enteroscopic biopsy.

Variables	Univariate Analysis		Multivariate Analysis	
	OR ^1^ (95% CI ^2^)	*p*-Value	OR (95% CI)	*p*-Value
Insertion route				
		0.005		0.838
Retrograde	0.33 (0.15–0.72)		1.13 (0.33–3.87)	
History of abdominal surgery				
		0.948		
No	0.96 (0.35–2.66)			
Final diagnosis				
		<0.001		0.004
Tumor disease	10.73 (3.90–29.51)		6.21 (1.80–21.43)	
Biopsy location				
		<0.001		0.138
Ileum/Multiple	0.21 (0.09–0.48)		0.34 (0.08–1.40)	
Biopsy number	1.36 (1.13–1.65)	0.001		

^1^ OR, odds ratio; ^2^ CI, confidential interval.

## Data Availability

The datasets generated or analyzed during the current study are available from the corresponding author on reasonable request.

## Supplementary Materials

The following supporting information can be downloaded at: https://www.mdpi.com/article/10.3390/diagnostics12040964/s1. Yields in the tumor and non-tumor group/s1, Supplementary Table S1.
