# Supplementary material for: Histological Diagnostic Yield and Clinical Significance of the First Biopsy in Device-Assisted Enteroscopy in Patients with Small Bowel Diseases: A KASID Multicenter Study
Source: Diagnostics (Basel). 2022 Apr 12;12(4):964. doi: 10.3390/diagnostics12040964 (PMC9031609; doi:10.3390/diagnostics12040964)
Supplement: Supplementary file 1 [file diagnostics-12-00964-s001.zip › diagnostics-1653366-supplementary.pdf]

Supplementary Table S1. Diagnostic yields in the tumor and non-tumor group

Diseases	Diagnostic (N = 49)	Non-diagnostic (N = 63)
Non-tumor, n (%)		
Crohn's disease	16 (27.1)	43 (72.9)
Intestinal tuberculosis	3 (30.0)	7 (70.0)
Eosinophilic enteritis	2 (66.7)	1 (33.3)
Behcet's disease	0	3 (100)
Vasculitis	1 (33.3)	2 (66.7)
Meckel diverticulum	1 (50.0)	1 (50.0)
Tumor, n (%)		
Adenocarcinoma	15 (100)	0
Lymphoma	9 (81.8)	2 (18.2)
Malignant GIST [1]	1 (50)	1 (50)
Inflammatory polyp	1 (100)	0
Lipoma	0	1 (100)
Leiomyosarcoma	0	2 (100)

[1] GIST, gastrointestinal stromal tumor
